# Prior testosterone use does not appear to impact oocyte cryopreservation outcomes in transgender patients: findings from a multicenter health maintenance organization

**DOI:** 10.1016/j.xfre.2025.06.011

**Published:** 2025-07-01

**Authors:** Natasha Raj-Derouin, Snunit Ben-Ozer, Amy Shah Dhesi, Tracy Harrison, Ashley Kim, Mandana Ghahremani, Sami Jabara, Marsha Baker

**Affiliations:** a Department of Obstetrics and Gynecology, Kaiser Permanente Los Angeles Medical Center, Los Angeles, California; b Department of Reproductive Endocrinology and Infertility, Kaiser Permanente Los Angeles Medical Center, Los Angeles, California; c Department of Reproductive Endocrinology and Infertility, Kaiser Permanente Woodland Hills Medical Center, Woodland Hills, California; d Department of Reproductive Endocrinology and Infertility, Kaiser Permanente San Diego Medical Center, San Diego, California; e Department of Reproductive Endocrinology and Infertility, Kaiser Permanente Downey Medical Center, Downey, California

**Keywords:** Transgender, fertility, preservation, testosterone, LGBTQIA+

## Abstract

**Objective:**

To compare the outcomes of oocyte cryopreservation in transgender patients with and without prior testosterone use

**Design:**

Retrospective cohort study

**Subjects:**

Transmasculine/non-binary patients assigned female at birth who were referred for fertility preservation from January 2012 to March 2024 at a multicenter health maintenance organization

**Exposure:**

Gender-affirming hormone therapy with testosterone

**Main Outcome Measures:**

The primary outcome measure was the number of mature oocytes collected. Secondary outcomes included the total number of oocytes, percentage of mature to total oocytes, total dose of gonadotropins used, baseline antimüllerian hormone, baseline antral follicle count, and baseline endometrial thickness.

**Results:**

Data from 50 transgender oocyte cryopreservation cycles were analyzed in the time period studied. A total of 34 subjects (68%) had no exposure to testosterone, whereas prior testosterone use was reported in 16 subjects (32%). The mean duration of testosterone was 4.1 ± 2.6 years. Testosterone was discontinued 3 weeks to 3 months before cycle start. There were no significant differences in baseline demographics, such as age and body mass index, between the two patient groups. The total number of oocytes retrieved was statistically the same between transgender patients with prior testosterone use (17.3 ± 10.1) and those without (21.3 ± 10.1). Additionally, there were no differences in the number of mature oocytes (12.0 ± 7.5 vs. 16.1 ± 9.1) or ratio of mature/total oocytes between the two groups (72.3% ± 18.3% vs. 70.9% ± 22.4%). Secondary outcomes such as baseline antral follicle count, baseline antimüllerian hormone, total dose of gonadotropins used, and baseline endometrial thickness demonstrated no significant difference between the two study groups.

**Conclusion:**

Our study suggests that prior testosterone use does not appear to impact oocyte cryopreservation outcomes in transgender patients. As a growing number of transgender patients seek fertility care, there is a need for evidence-based research that can guide clinical practice and empower this population to realize their aspirations for parenthood.

Approximately 1.6 million people in the United States identify as transgender (1). Identifying as transgender broadly signifies an individual whose gender identity does not match the sex they were assigned at birth (2). Many of these individuals experience gender dysphoria, which refers to the psychological distress caused by the incongruence of one’s gender identity and their assigned sex at birth (3). Gender-affirming treatments, such as hormonal therapy and surgery, have been shown to greatly reduce gender dysphoria (4).

Of the transgender individuals in the United States, 36% identify as transgender men and 26% identify as gender non-binary (1). Transgender and non-binary individuals who were assigned female at birth may take gender-affirming hormone therapy (GAHT) such as exogenous testosterone and puberty blockers, as well as pursue surgical management such as hysterectomy, salpingectomy, oophorectomy, chest surgery, and genital surgery (5).

Studies have shown that over 50% of transgender individuals have a desire for future children, with nearly 40% desiring biological children (6). However, limited research exists regarding the effect of GAHT, such as testosterone, on fertility potential. The historic understanding of testosterone’s effects on ovarian tissue largely stems from histologic analyses of surgically removed ovaries from transgender men after gender-affirming surgery (7, 8, 9). These studies have consistently demonstrated that testosterone exposure is associated with polycystic ovarian syndrome-like morphology, with changes in ovarian specimens seen, such as stromal luteinization, increased collagenization, and multiple cystic follicles (9, 10, 11). Thus, it is posited that exogenous testosterone may be associated with subfertility or infertility through similar mechanisms as seen with polycystic ovarian syndrome.

Throughout the nation, new laws are being passed that require insurers to provide coverage for fertility preservation (FP) for iatrogenic infertility, such as infertility caused by GAHT or surgery (12). As of January 2025, nineteen states have enacted insurance mandates for FP coverage (13). As accessibility and affordability of these services improve, an increasing number of transgender patients will seek out reproductive and fertility care, rendering the need for additional research on this matter. The objective of this study was to compare the outcomes of oocyte cryopreservation in transgender patients with and without prior testosterone use.

## Materials and methods

### Study design

This is a retrospective cohort study of transgender/non-binary patients assigned female at birth who were referred for FP from January 2012 to March 2024 at a multicenter health maintenance organization. This study was performed in a state that passed a mandate for insurance coverage of FP services for transgender patients on January 1, 2020. Data were extracted from a centralized electronic medical record system of 10 regional hospitals within the health maintenance organization. Inclusion criteria encompassed patients assigned female at birth who identify as transgender or gender-nonbinary. Exclusion criteria included patients who were assigned male at birth. Patients were identified using the ICD-10 code “Encounter for Fertility Preservation Counseling” and included in the study if they underwent oocyte cryopreservation. All transgender patients referred for FP followed a standardized workflow within our health maintenance organization. After initial referral, patients attended a comprehensive consultation with a reproductive endocrinology and infertility (REI) specialist where they received detailed counseling about FP options. Patients who elected to pursue FP then underwent diagnostic work-up, including laboratory evaluation (estradiol, antimüllerian hormone [AMH], and follicle-stimulating hormone) and transvaginal ultrasound for antral follicle count and endometrial thickness assessment. After completion of the diagnostic work-up, patients who remained interested proceeded with ovarian stimulation and oocyte retrieval. Each REI physician in the health maintenance network was invited to participate in this study and provide data regarding the oocyte cryopreservation outcomes. Seven physicians agreed to participate, resulting in 50 patients included in the study. These patients were then divided into the study and control group, the study group being transgender patients currently on testosterone and transgender patients not on testosterone.

This study was approved by the Institutional Review Board of the Kaiser Permanente Los Angeles Medical Center, with a waiver of consent for retrospective analysis of deidentified data.

### Study outcomes

Baseline demographic data were collected on each patient’s age, race/ethnicity, body mass index (BMI, kg/m^2^), and smoking history. Additional data on prior testosterone use, duration of use, and time of discontinuation before cycle start were collected. The primary outcome measure was the number of mature oocytes in meiosis II (MII) that were collected. Secondary outcomes included the total number of oocytes, percentage of MII to total oocytes (%), total units of gonadotropins used during stimulation (IU), baseline AMH (ng/dL), baseline antral follicle count (AFC), and endometrial thickness (mm).

### Ovarian stimulation protocol

All patients in the study underwent initial fertility evaluation with a REI specialist within the health maintenance network. Initial work-up included laboratory testing for estradiol, AMH, and follicle-stimulating hormone. Ultrasound imaging on day 2 or 3 of the patient’s menstrual cycle was used to determine the starting AFC and endometrial thickness. A gonadotropin-releasing hormone (GnRH) antagonist-controlled ovarian stimulation protocol was used for all patients. Some patients received clomiphene citrate or letrozole in addition to gonadotropins for controlled ovarian stimulation. Ovarian follicle growth was monitored using transabdominal or transvaginal ultrasound. Ovulation was triggered with human chorionic gonadotropin, leuprolide acetate, or a combination of both. Oocyte retrieval occurred 35 hours after the trigger.

### Statistical analysis

Continuous variables were compared using a two-tailed *t*-test, and categorical variables were compared using a χ^2^ test. Additionally, multiple linear regression analysis was performed to examine the relationship between prior testosterone use and number of mature oocytes while controlling for potential confounders (age, BMI, baseline AMH, and baseline AFC). A statistically significant value was defined as a *P* value less than .05.

A subanalysis of the transgender patients on testosterone therapy was performed to explore potential relationships between testosterone therapy parameters and oocyte yield. Spearman’s rank correlations were conducted to examine associations between duration of testosterone therapy, washout period (time since last testosterone injection), total oocytes retrieved, and number of MII retrieved among the subset of patients with prior testosterone exposure. This non-parametric approach was selected as appropriate for the sample size of this subgroup analysis.

## Results

**Figure 1:**
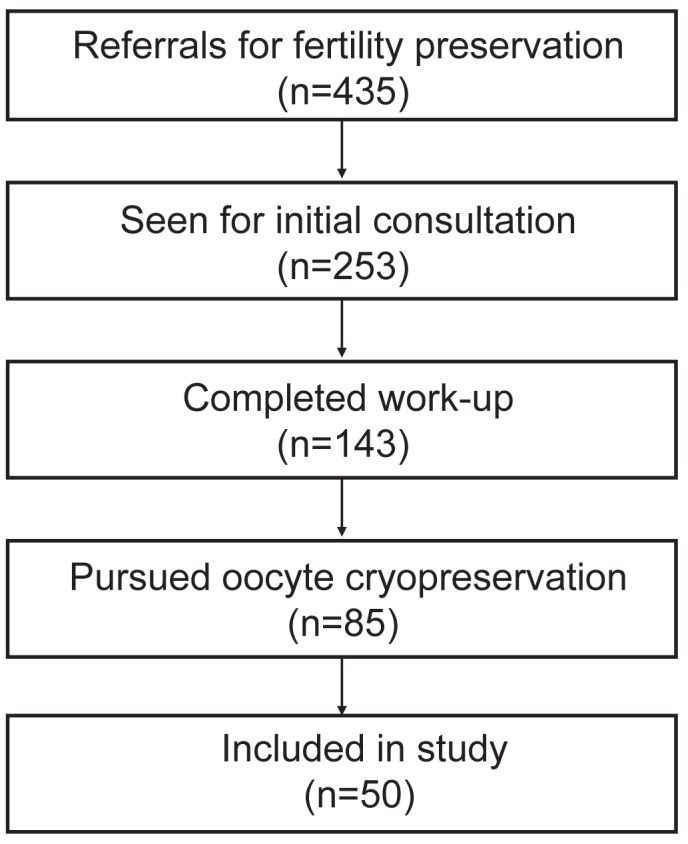
Flow chart of patient selection. During the study period, 435 transgender and gender-diverse individuals who were assigned female at birth were referred for fertility preservation. Of those patients, 253 (58%) attended an initial consultation with an REI physician; 143 (33%) patients completed the diagnostic work-up, including laboratories and ultrasound imaging; 85 (20%) pursued oocyte cryopreservation, and 50 (11%) were included in this study. REI = reproductive endocrinology and infertility.

Between January 2012 and March 2024, 435 FP referrals were placed for transgender and non-binary patients assigned female at birth. Of those referrals, 143 patients completed a work-up, and 85 of those patients completed an FP cycle (Fig. 1). Fifty patients were ultimately included in this study after excluding patients whose charts were incomplete or contained missing data (n = 35). Of our study sample of 50 transgender patients, 34 patients (68%) had no exposure to testosterone before their FP cycle. Prior testosterone use was reported in 16 subjects (32%). The mean duration of prior testosterone use was 4.1 ± 2.6 years. Testosterone was discontinued between 3 weeks to 3 months before cycle start based on the REI physician's discretion. All patients had reached menarche before pursuing FP, and only two patients underwent pubertal suppression with a GnRH agonist before FP, one who had no prior testosterone use and one with testosterone use.

There were no significant differences in baseline demographics, such as age at FP, race/ethnicity, BMI, and smoking history, between those with and without prior testosterone (T) exposure (Table 1). The two groups were on average the same age (prior T use 25.1 ± 4.6 vs. no T use 22.3 ± 8.0 years, *P*=.13). However, the proportion of patients aged equal to or less than 18 was lower in the testosterone-exposed group in comparison with the control testosterone-naive group (Table 1). Additionally, there was a higher proportion of patients aged 19–30 in those with prior testosterone use than in those without. Patients of both groups had a similar average BMI (prior T use 27.9 ± 6.3 vs. no T use 26.9 ± 9.0 kg/m^2^, *P*=.68). The composition of racial/ethnic backgrounds was statistically similar between the two study groups (Table 1). There was no statistically significant difference in the percentage of patients with a previous smoking history (prior T use 19% ± 0.4% vs. no T use 6% ± 0.2%, *P*=.25).

**Table 1:** Demographic characteristics of transgender patients with and without prior testosterone use.

Characteristics	With prior T use (n = 16)	No T use (n = 34)	*P* value
Age (y)	25.1 ± 4.6	22.3 ± 8.0	.13
Age <18	2/16 (12.5%)	15/34 (44%)	.03
Age 19–30	12/16 (75%)	12/34 (35.3%)	.008
Age 31–35	2/16 (12.5%)	5/34 (14.7%)	.83
Age 36+	0/16 (0%)	2/34 (5.9%)	NA
BMI (kg/m^2^)	27.9 ± 6.3	26.9 ± 9.0	.68
Race and ethnicity			.78
White	5/16 (31.3%)	14/34 (41.2%)	.49
Black	1/16 (6.2%)	4/34 (11.8%)	.54
Asian	1/16 (6.2%)	3/34 (8.8%)	.75
Hispanic/Latinx	8/16 (50%)	10/34 (29.4%)	.16
American Indian/Pacific Islander	0/16 (0%)	1/34 (2.9%)	NA
Declined/Other	1/16 (6.2%)	2/34 (5.9%)	.96
History of smoking (%)	19% ± 0.4%	6% ± 0.2%	.25

*Note:* Data reported as mean ± SD or as percentage. BMI = body mass index; NA = not applicable; T = testosterone.

The total number of oocytes retrieved was statistically the same between transgender patients with prior testosterone use (17.3 ± 10.1) and those without (21.3 ± 10.1, *P*=.20) (Table 2). Additionally, there were no differences in number of MII (prior T use 12.0 ± 7.5 vs. no T use 16.1 ± 9.1, *P*=.10) or ratio of MII/total oocytes between the two groups (prior T use 72.3% ± 18.3% vs. no T use 70.9% ± 22.4%, *P*=.81) (Table 2). Secondary outcomes such as baseline AFC, baseline AMH, total dose of gonadotropins used, and baseline endometrial thickness demonstrated no significant difference between the two study groups (Table 3).

**Table 2:** Primary outcomes between transgender patients with and without prior testosterone use.

Outcomes	With prior T use (n = 16)	No T use (n = 34)	*P* value
Total number of oocytes	17.3 ± 10.1	21.3 ± 10.1	.20
Number of MII	12.0 ± 7.5	16.1 ± 9.1	.10
Ratio of MII/total oocytes (%)	72.3% ± 18.3%	70.9% ± 22.4%	.81

*Note:* Data reported as mean ± SD or as percentage. MII = mature oocytes in meiosis II; T = testosterone.

**Table 3:** Secondary outcomes between transgender patients with and without prior testosterone use.

Outcomes	With prior T use (n = 16)	No T use (n = 34)	*P* value
Baseline AFC (n)	23.4 ± 11.3	25.3 ± 16.5	.66
Baseline AMH (ng/dL)	3.6 ± 3.0	4.3 ± 2.5	.46
Baseline FSH (mIU/mL)	4.6 ± 2.4	4.7 ± 1.7	.92
Endometrial lining (mm)	4.9 ± 2.0	5.9 ± 2.6	.13
Total gonadotropins used (IU)	3064.3 ± 984.3	3028.1 ± 909.8	.90
Peak E_2_ serum level (pg/mL)	2004.9 ± 2564.8	2500.9 ± 1592.1	.49

*Note:* Data reported as mean ± SD. AFC = antral follicle count; AMH = antimüllerian hormone; FSH = follicle-stimulating hormone; T = testosterone.

To further examine the impact of prior testosterone use on oocyte yield while controlling for potential confounding variables, we performed multiple linear regression analysis. After adjusting for age, BMI, baseline AMH, and baseline AFC, prior testosterone use was not significantly associated with the number of mature oocytes retrieved (B = −0.803, 95% confidence interval [−5.546, 3.939], *P*=.733). Age was the only significant predictor of oocyte yield in our model (B = −0.530, 95% confidence interval [−0.884, −0.176], *P*=.004). The overall model explained 42.8% of the variance in mature oocyte yield (R^2^ = 0.428, F (5,35) = 5.245, *P*=.001).

In the subgroup of patients with prior testosterone use (n = 16), we conducted exploratory analyses examining correlations between testosterone therapy parameters and fertility outcomes. No statistically significant correlations were found between years on testosterone therapy and number of oocytes retrieved (rs = −0.046, *P*=.866), number of mature oocytes (rs = −0.164, *P*=.544), or percentage of mature oocytes (rs = −0.391, *P*=.135). Similarly, the washout period (time since last testosterone dose) did not significantly correlate with the number of oocytes retrieved (rs = −0.155, *P*=.648), number of mature oocytes (rs = −0.156, *P*=.648), or percentage of mature oocytes (rs = 0.083, *P*=.809).

## Discussion

Recent legislative changes mandating insurance coverage for FP services have significantly expanded access to reproductive healthcare for transgender individuals pursuing GAHT. As more transgender patients seek FP services, fertility care providers face critical decisions regarding the optimal timing of oocyte cryopreservation in relation to initiating testosterone therapy. Although existing guidelines recommend FP counseling before starting GAHT, there is limited empirical evidence on clinical recommendations about whether previous testosterone exposure impacts oocyte cryopreservation outcomes. Our findings suggest that prior testosterone therapy does not significantly affect oocyte cryopreservation outcomes in transgender patients.

Our work builds on a growing body of clinical research about the effect of testosterone on fertility in transgender and gender-diverse patients. Most of the clinical research published in this arena has been limited to case series and matched cohort studies with small sample sizes. Several studies compared transgender men both with and without testosterone exposure to a matched cohort of cisgender women and found no differences in oocyte yield during cryopreservation (14, 15). Additionally, Israeli et al. (16) compared preimplantation embryo quality of transgender patients on testosterone vs. a cisgender matched cohort and demonstrated a similar fertilization rate and morphologic score of embryos between the two groups. A 2023 descriptive study of 16 transgender men, 11 of whom were on testosterone before fertility treatments, were able to successfully undergo oocyte cryopreservation, IVF, pregnancy, and live birth (17). Other smaller cohort studies have been performed that directly compared transgender men without testosterone exposure with transgender men with prior testosterone use and found no difference in estradiol level per oocyte, total oocyte yield, and maturity rate (18). Our findings not only support current literature that testosterone therapy does not compromise FP outcomes in transgender patients, but strengthen the collective data on this patient population from our larger sample sizes.

Our exploratory subanalysis examining correlations between testosterone therapy parameters and oocyte yield revealed no statistically significant associations between duration of testosterone therapy or washout period and oocyte yield or maturity rate. This finding, although not statistically significant, requires cautious interpretation because of our limited sample size. It also highlights the importance of further research into how specific testosterone parameters might influence FP outcomes. Future studies with larger cohorts should be adequately powered to detect potential relationships between testosterone duration, dosage, washout protocols, and reproductive outcomes, which could help optimize FP timing and protocols for transgender patients.

Although standard practice typically involves discontinuing testosterone therapy before ovarian stimulation, emerging evidence suggests this may not be an absolute requirement. Although most published studies have included testosterone cessation protocols, select case reports have demonstrated the feasibility of continuing testosterone therapy during ovarian stimulation. One case report described a successful oocyte retrieval of 22 mature oocytes in a transgender man who had been on testosterone therapy for 18 months without interruption, whereas another described a transgender man who had been on testosterone for 3 years and discontinued it for only three doses (immediately before and during stimulation) before retrieval of 11 mature oocytes (19, 20). Although these reports are limited by small sample sizes and preclude definitive conclusions about safety and efficacy, they open important discussions about potentially modifying current testosterone discontinuation protocols and further indicate that testosterone may not affect oocyte yield. This consideration is particularly relevant given that the requirement to stop testosterone therapy represents a significant barrier for many transgender men considering FP, sometimes leading to the decision to forego this option entirely.

The perception that testosterone therapy may negatively impact fertility potential often influences the timing of when patients initiate GAHT in relation to FP. This is reflected in our finding that the testosterone-naive group had a significantly higher proportion of patients aged equal to or less than 18 compared with the testosterone-exposed group (44% vs. 12.5%, *P*=.03). Although this difference could be partially attributed to the natural relationship between age and medication exposure time, it may also reflect a bias in how younger patients are counseled in multidisciplinary transgender clinics regarding the optimal timing of GAHT initiation in relation to FP. Notably, despite the testosterone-naive group having a higher proportion of patients aged equal to or less than 18, there were no significant differences in oocyte yield between the groups. This finding is particularly compelling given that younger age is typically associated with higher oocyte yield in FP cycles, further supporting our conclusion that testosterone exposure does not adversely affect oocyte cryopreservation outcomes in transgender patients.

This study has several strengths, including that this is the largest reported cohort of transgender men undergoing FP to date. Our ability to achieve this substantial cohort size was facilitated by California’s insurance mandate requiring coverage of FP for transgender patients, which significantly improved access to these services (21). Furthermore, our health maintenance organization coordinates multidisciplinary care for transgender patients, allowing easy referral to REI for any transgender patient seeking FP, regardless of which department the patient was initially receiving care. The multicenter nature of our data collection further strengthens the generalizability of our results across different clinical settings and patient populations. Additionally, our study design directly compares outcomes between testosterone-exposed and testosterone-naive transgender men, rather than utilizing a matched cisgender female control group, which reduces potential confounding variables.

This study has several limitations that warrant consideration. First, the retrospective design inherently introduces potential biases and limits our ability to control for confounding variables. Additionally, there was notable heterogeneity in the timing of testosterone discontinuation before ovarian stimulation, which may impact the interpretation of our findings. The variation in discontinuation periods (3 weeks to 3 months) primarily reflects differences in individual provider preferences based on their clinical judgment, evolving literature, and patient-specific factors. Although this heterogeneity is a limitation of our study, it also reflects the real-world clinical practice environment where evidence-based guidelines for transgender fertility care are still developing. Furthermore, although we were able to assess immediate markers of FP success such as oocyte yield and maturity rate, the long-term reproductive potential of cryopreserved oocytes from this population remains unclear. As very few transgender patients have returned to use their cryopreserved oocytes, data on pregnancy and live birth outcomes are limited, highlighting the need for longer-term follow-up studies in this population.

Finally, although this study represents the largest cohort of transgender men undergoing FP reported to date, the sample size remains relatively small. Post-hoc power analysis revealed that our study was adequately powered (>80%) to detect large effect sizes but had limited power to detect smaller effects. Our findings should be interpreted conservatively as preliminary evidence that large, clinically meaningful differences in oocyte yield are unlikely with prior testosterone exposure, although smaller effects cannot be ruled out. Larger studies are needed to draw more generalizable conclusions about the impact of testosterone exposure on FP outcomes. The smaller sample size can be partially attributed to the attrition seen throughout the FP process. Very few patients who were seen for FP consultation completed the process, as illustrated in Figure 1. Although we do not have systematic data on the exact reasons for discontinuation of care, chart review provided insights into common barriers. Frequently cited reasons in clinical notes included reluctance to delay initiation of testosterone if not already on it, gender dysphoria potentially exacerbated by oocyte-stimulating medications, and discomfort with the invasiveness of transvaginal ultrasounds. Our findings may help address some of these barriers by demonstrating that testosterone does not appear to meaningfully impact oocyte cryopreservation outcomes, potentially allowing patients to pursue FP at a later time after starting testosterone therapy. Additional interventions to improve uptake could include prioritizing transabdominal ultrasound monitoring when clinically appropriate, incorporating medications such as letrozole into stimulation protocols to mitigate estrogen-related effects, and developing more transgender-affirming counseling approaches that address the unique psychological considerations this population faces during FP procedures.

There are many future directions that research on fertility care for transgender and gender-diverse patients can expand upon. First, larger studies are needed to further evaluate the relationship between GAHT and fertility, particularly examining the impact of duration on testosterone and optimal timing of discontinuation before ovarian stimulation. Long-term follow-up studies can assess embryologic development and pregnancy outcomes from oocytes retrieved after testosterone exposure, as current data on live birth rates remains limited. Given the increasing number of adolescent patients seeking gender-affirming care, targeted research examining testosterone's effects on fertility in this younger population would be particularly valuable to guide evidence-based counseling regarding the timing of FP in relation to GAHT initiation. Such research would help clinicians provide more precise recommendations about the optimal sequencing of these interventions while supporting patients in achieving both their gender-affirming and reproductive goals.

## Conclusion

Our study suggests that prior testosterone use does not appear to impact oocyte cryopreservation outcomes in transgender patients with similar total oocyte yield, maturity rate, and total dose of gonadotropins used. Additional studies are needed to investigate the impact of duration on testosterone and the length of discontinuation before ovarian stimulation on subsequent reproductive outcomes. As a growing number of transgender patients seek fertility care, there is a need for more evidence-based research that can guide clinical practice, further empowering this population to realize their aspirations for parenthood.

## CRediT Authorship Contribution Statement

**Natasha Raj-Derouin:** Writing – review & editing, Writing – original draft, Visualization, Validation, Project administration, Methodology, Investigation, Formal analysis, Data curation, Conceptualization. **Snunit Ben-Ozer:** Writing – review & editing, Data curation, Conceptualization. **Amy Shah Dhesi:** Writing – review & editing, Data curation, Conceptualization. **Tracy Harrison:** Writing – review & editing, Data curation. **Ashley Kim:** Writing – review & editing. **Mandana Ghahremani:** Writing – review & editing, Data curation. **Sami Jabara:** Writing – review & editing, Supervision, Data curation, Conceptualization. **Marsha Baker:** Writing – review & editing, Supervision, Project administration, Methodology, Data curation, Conceptualization.

### Declaration of Interests

N.R.-D. has nothing to disclose. S.B.-O. has nothing to disclose. A.S.D. has nothing to disclose. T.H. has nothing to disclose. A.K. has nothing to disclose. M.G. has nothing to disclose. S.J. has nothing to disclose. M.B. has nothing to disclose.

### Declaration of Generative AI and AI-Assisted Technologies in the Writing Process

During the preparation of this work the authors used Claude.AI in order to edit the writing language and improve readability. After using this tool/service, the authors reviewed and edited the content as needed and took full responsibility for the content of the published article.
